# Cepharanthine inhibits hepatocellular carcinoma cell growth and proliferation by regulating amino acid metabolism and suppresses tumorigenesis *in vivo*

**DOI:** 10.7150/ijbs.64675

**Published:** 2021-10-22

**Authors:** Fan Feng, Lianhong Pan, Jiaqin Wu, Lanqing Li, Haiying Xu, Li Yang, Kang Xu, Chunli Wang

**Affiliations:** 1 National Innovation and Attracting Talents “111” base, Key Laboratory of Biorheological Science and Technology, Ministry of Education, College of Bioengineering, Chongqing University, Chongqing 400030, China.; 2 Chongqing Engineering Research Center of Antitumor Natural Drugs, Chongqing Three Gorges Medical College, Chongqing 400030, China.; 3 Hubei Engineering Technology Research Center of Chinese Materia Medica Processing, College of Pharmacy, Hubei University of Chinese Medicine, Wuhan 430065, China.

**Keywords:** Cepharanthine, Hepatocellular carcinoma, Metabolism, Apoptosis.

## Abstract

Cepharanthine (CEP), a natural compound extracted from *Stephania cepharantha* Hayata, has been found to have the potential to treat a variety of tumors in recent years. This study aims to evaluate the anti-hepatocellular carcinoma (HCC) effect of CEP and determine its in-depth mechanism. In this study, Hep3B and HCCLM3 cells were selected to evaluate the antitumor effects of CEP *in vitro*, whereas tumor xenograft in nude mice was performed to make *in vivo* anti-tumor assessment. RNA-sequence (RNA-seq) was used to identify possible molecular targets and pathways. Further, gas chromatography mass spectrometry (GC-MS) was performed to assess the differential metabolites involved in mediating the effect of CEP on the HCC cell line. Our results showed that CEP treatment resulted in the dose-dependent inhibition of cell viability, migration, and proliferation and could also induce apoptosis in HCC cells. RNA-seq following CEP treatment identified 168 differentially expressed genes (DEGs), which were highly enriched in metabolism-associated pathways. In addition, CEP down-regulated many metabolites through the amino acid metabolism pathway. *In vivo* experiment showed that CEP significantly suppressed tumor growth. Our results indicate that CEP has significant antitumor effects and has the potential to be a candidate drug for HCC treatment.

## Introduction

Hepatocellular carcinoma (HCC) is the most common type of primary liver cancer with a high morbidity and mortality rate, and seriously endangers human health 1. According to statistics, the number of liver cancer-related deaths in 2020 reached 830,000, accounting for 8.3% of the world's cancer-related deaths 2. Because HCC is diagnosed only in the middle and advanced stages, the chances of radical treatments such as surgery or liver transplantation are lost 3. As a result, chemotherapy has become a common treatment option 4. Currently, there are only a few commonly used chemotherapy drugs for liver cancer, such as sorafenib 5, cisplatin 6, 5-fluorouracil 7, doxorubicin 8, and oxaliplatin 9. Cisplatin (CDDP) is one of the conventional chemotherapy drugs, widely used in melanoma and lung, ovarian, liver, and other solid tumors. CDDP can lead to cell necrosis and apoptosis by causing DNA damage and then activating several transduction pathways 10, 11. However, the efficacy of CDDP in treating liver cancer is not ideal; there are some shortcomings such as high toxicity and strong drug resistance 12. Therefore, it is necessary to develop new drugs and targets for the treatment of HCC.

Cepharanthine (CEP) is a natural small molecule alkaloid extracted from the plant *Stephania cepharantha* Hayata, which has multiple pharmacological properties such as anti-inflammatory, antioxidant, and immunoregulatory properties without major side effects 13, 14. CEP also has various anticancer effects, including inhibition of cell proliferation, promotion of cell apoptosis, and anti-angiogenesis 15, 16. Recent studies have shown that CEP damages the DNA of lung cancer cells by promoting the production of reactive oxygen species, leading to the death of lung cancer cells and cell cycle arrest 17. CEP can induce the apoptosis of human leukemia T cells by upregulating the expressions of caspase-8, 9; caspase-3, 6; and other effector caspases 18. CEP can also inhibit breast cancer cells by inducing autophagy 19. Rattanawong et al reported that CEP could induce cell cycle arrest and apoptosis through the upregulation of p21Waf1/Cip1 and downregulation of cyclin A and Bcl2 in p53 mutant colorectal cancer cells 20. Abnormal metabolism is an important characteristic of HCC. The rapid proliferation of HCC cells relies on a series of metabolic reprogramming reactions to provide energy and raw materials 21. Therefore, this study aimed to investigate whether CEP can inhibit the proliferation of HCC and determine its in-depth mechanism, as well as investigate whether CEP can regulate the metabolism of HCC cells.

In this study, inhibition of cell viability, migration, and proliferation was observed in HCC cells treated by CEP. The inhibition, migration, and proliferation were observed in the HCC cells treated by CEP. Further experiments demonstrated that CEP treatment also promoted the apoptosis of HCC cells by activating caspase protease. Metabonomic results indicated that CEP down-regulates many metabolites through the amino acid metabolism pathway. In addition, subcutaneous tumor formation experiments in nude mice confirmed that CEP could significantly inhibit the growth of solid tumors. These results suggested that CEP has the potential to be a candidate drug for HCC treatment.

## Materials and methods

### Cell culture and reagents

The human hepatica cell lines including Hep3B and HCCLM3 and human normal liver cell lines L02 were obtained from the Shanghai Cell Bank (China, Shanghai). The cells were cultured in DMEM medium (Gibco, USA) containing 10% fetal bovine serum (FBS; Gibco, USA) and 1% penicillin-streptomycin (Gibco, USA) under the condition containing 5% 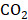 at 37 °C. CEP, supplied by Selleck Chemicals (Houston, USA), was dissolved in dimethyl sulfoxide (DMSO; SIGMA, USA) to obtain a 10 mM stock solution, which was then diluted with autoclaved DMEM medium to use.

### Cell viability analysis

A total of 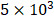 Hep3B, HCCLM3 and L02 cells per well were placed into the 96-well plates with three wells used for each treated group. Cells were serum starved for 12 h and then incubated with different concentrations of CEP for 48 h. Cell viability was monitored with a cell counting kit-8 (CCK-8; Dojindo, Japan). Ten microliters of CCK-8 reagent were mixed with cells and incubated for 1 h 30 min. The final absorbance at 450 nm was evaluated in each well by use of a MultiSkan GO microplate reader (Thermo, USA).

### Cell cycle analysis

After serum starvation for 12 h, the cells were treated with 5 µM, 10 µM and 20 µM of CEP, 0.2% DMSO (vehicle control) for 48 h. After incubation, cells were harvested by trypsinization and the cell pellets were fixed with cold 75% ethanol at 4 °C overnight. Cell staining was performed by incubating samples with PI for 15 min at room temperature in the dark. DNA contents of the stained cells were analyzed using a flow cytometer (BD Bioscience).

### Apoptosis assay

After treated with 5 µM, 10 µM and 20 µM of CEP for 48h, cells were harvested by trypsinization and the cells were centrifuged for 5 min in pre-cooled phosphate buffer (PBS) to wash the cells for two times. Then the cells were incubated with annexin V-FITC and PI for 20 min at room temperature in the dark. Before operating the machine, 400 μL buffer was added and the apoptosis rate was measured by a flow cytometer. CDDP (2 μg/mL) treatment were used as positive control.

### Clone formation assay

In the culture medium, the cells in the logarithmic growth phase were seeded with 500 cells per well and cultured in 6-well plates for 5 days. Then the fresh medium with different concentrations of CEP was replaced. After 5 days of continuous culture, the cell colonies were fixed with 4% paraformaldehyde at room temperature for 1h. After stained with 0.05% crystal violet for 10 min, the cell colonies were photographed and counted.

### *In vitro* migration assays

Cell scratch assay was used to detect the effect of CEP on the migration ability of hepatoma cells *in vitro*. In brief, Hep3B and HCCLM3 were first planted on a 6-well plate. When the cells grew to 70% density, the fused monolayer cells were scratched with a 200 μL sterile pipette and washed twice with PBS to remove cell debris. Then Serum-free medium was added to the wells with different concentrations of CEP. After incubation for 48 h at 37 °C, the cells were fixed with 4% paraformaldehyde at room temperature for 1 h, and then it was stained with 0.05% crystal violet for 5 min. After washed with 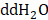 for two times, the scratch images were taken using a microscope.

### Western Blotting

Hep3B and HCCLM3 cells were collected and lysed in RIPA protein extraction reagent (Beyotime, Beijing, China). The enhanced BCA Protein assay kit (Beyotime, Beijing, China) was used to quantify the protein concentration. Equal amounts of protein were dissolved in 10% SDS-PAGA and then transferred to PVDF membranes overnight. After sealing with 5% skim milk powder for 1 h, the membranes were incubated overnight with the following primary antibody at 4 °C. Bax, Bcl2, myc, surviving, C-PARP, cleaved-Caspase 3, cleaved-Caspase 9 and Tubulin. The membranes were then washed and incubated with HRP-linked secondary antibody for 1 h at room temperature. Finally, the samples from three independent experiments were observed by enhanced chemiluminescence (ECL, Thermo Scientific, USA). The band density was quantified using Image J software.

### Real time RT-PCR and RNA sequencing

Total RNA was extracted from Hep3B and HCCLM3 cells which treated with 5 µM, 10 µM and 20 µM of CEP by using TRIzol Reagent (Invitrogen, Thermo Scientific, USA). Partly isolated RNA samples were submitted to BGI Co., LTD for transcriptome sequencing and analysis on BGISEQ-500. And then cDNA was synthesized using PrimeScript™ RT reagent Kit (TaKaRa, Tokyo, Japan) according to the manufacturer's protocol. The PCR amplification was conducted using SYBR Premix Ex Taq™ II (TaKaRa, Tokyo, Japan). All the primer sequences were as Table 1. The gene expression levels for each amplification were calculated using the ΔΔCT method and normalized against GAPDH mRNA and all reactions were run in triplicate.

### Metabolite extraction and gas chromatography mass spectrometric (GC-MS) analysis

HCCLM3 cells were planted on a 6-well plate and treated with 20 µM CEP for 48 h. Following that, cells were quickly rinsed twice with precooled PBS. The well plate was transferred to ice, and 250 µL of -80 °C precooled methanol solution containing (10 µL/mL) was added. Liquid nitrogen was used to freeze the cells for 10 min, and then thawed for 5 min at room temperature. This process was repeated three times, and then centrifuged at 4 °C, the supernatant was transferred to a new EP tube and dried with a nitrogen blower at 35 °C. The dry samples were dissolved in pyridine with 80 µL methoxamine hydrochloride (20 mg/mL), and then eddy centrifuged for 2min and incubated at 37 °C for 2 h. Next, 50 μL BSTFA was added and eddy centrifuged for 2min. For complete derivatization, the samples were incubated in a water bath at 80 °C for 1h and then transferred to 200 μL micro-inserts. Trace1300 GC-MS System (Thermo, USA) was employed for the metabolomic analysis, along with computer-aided Similarity Evaluation System for Chromatographic Fingerprint of TCM (Version 2012) and SIMCA software (Umetrics, Sweden). The control group was treated with DMSO, and 8 samples were collected from each group.

### Assay of antitumor efficacy *in vivo*

20 female nude mice were purchased from Animal Experimental Center of Chongqing Medical University for subcutaneous tumorigenesis experiment. Animal experiments were approved by the Ethics Committee of Chongqing Medical University. Each nude mouse was subcutaneously injected with 2× 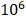 Hep3B cells in logarithmic growth phase in the right axilla. When the longest axis of the tumor was between 5 and 8 mm, the mice were randomly divided into control group, positive control group (Cisplatin, 5 mg/kg), low-concentration treatment group (CEP, 10 mg/kg) and high-concentration treatment group (CEP, 20 mg/kg), with 5 mice in each group. The drug was injected intraperitoneally every two days and the longest axes (L) and vertical axes (R) of the tumor were recorded. Tumor volumes (V) were reckoned using the following formula: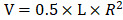. After treatment for 24 days, the nude mice were sacrificed, and the tumor tissues were harvested, then fixed overnight in 4% paraformaldehyde for subsequent immunohistochemical experiments.

### Histological analysis

For hematoxylin and eosin (H&E) staining, allograft tumors were fixed overnight in 4% paraformaldehyde. After dehydration, the samples were embedded in paraffin, then sliced into 6um thick sections using a cryostat (Leica, Wetzlar, Germany) and stained with H&E. For the immunohistochemical assay, after a standardized procedure, tissue sections were exposed to primary antibody Ki67 and incubated overnight. After washing PBS for three times, it was incubated with the secondary antibody at 37 °C for 30min, and then 3,3'-Diaminobenzidine (DAB) staining showed that the Ki67-positive cells were brown.

### Statistical analysis

Data were collected from three independent experiments and expressed as mean ± standard deviation (SD). All significance analyses, including one-way analysis of variance (ANOVA), multiple comparisons, and t-test, were performed using Origin software. P values were considered significant at 0.05 levels.

## Result

### CEP inhibits Hep3B and HCCLM3 cell viability in a concentration-dependent manner

**Figure 1:**
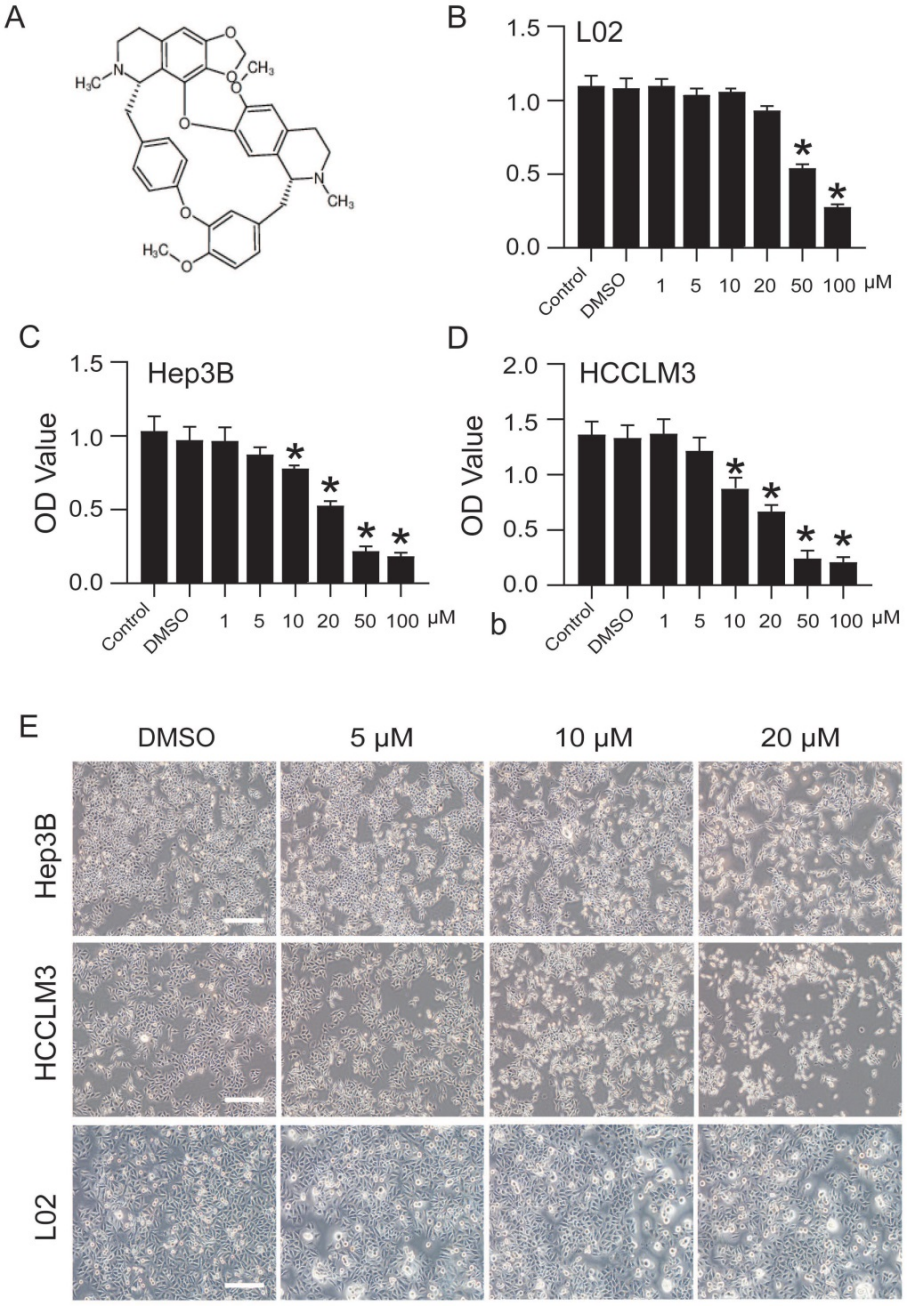
CEP inhibits Hep3B and HCCLM3 cells viability in a concentration dependent manner. (A) The molecular structure of CEP. (B-D) Cell viability of L02, Hep3B and HCCLM3 cells treated with CEP at indicate concentration was determined by CCK8 method, DMSO was used as control. (E) The morphology of Hep3B and HCCLM3 cells treating with CEP in different concentration of 5, 10 and 20 μM for 48 h. DMSO was used as control, scale bar=100 μm. Data shown are means ± SD; n=3. *P< 0.05, significantly from control group.

The structure of CEP (MW, 606.71) used in this study is shown in Fig. 1A. The effects of CEP on the viability of Hep3B, HCCLM3, and L02 cells were detected by CCK-8 assay. The results showed that CEP treatment for 48 h significantly inhibited the viability of Hep3B and HCCLM3 cell lines in a dose-dependent manner, and the 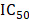 value was approximately 20 µM (Fig. 1C and D). Further, morphological changes in cells were observed through a microscope, and the results showed that with an increase in CEP concentration, the number of exfoliated cells gradually increased, and the number of adherent cells gradually decreased, indicating a concentration-dependent relationship (Fig. 1E). However, CEP concentrations of 10 and 20 µM showed no obvious cytotoxicity to the normal liver cell line L02 (Fig. 1B and 1E).

### CEP suppresses Hep3B and HCCLM3 cell proliferation

**Figure 2:**
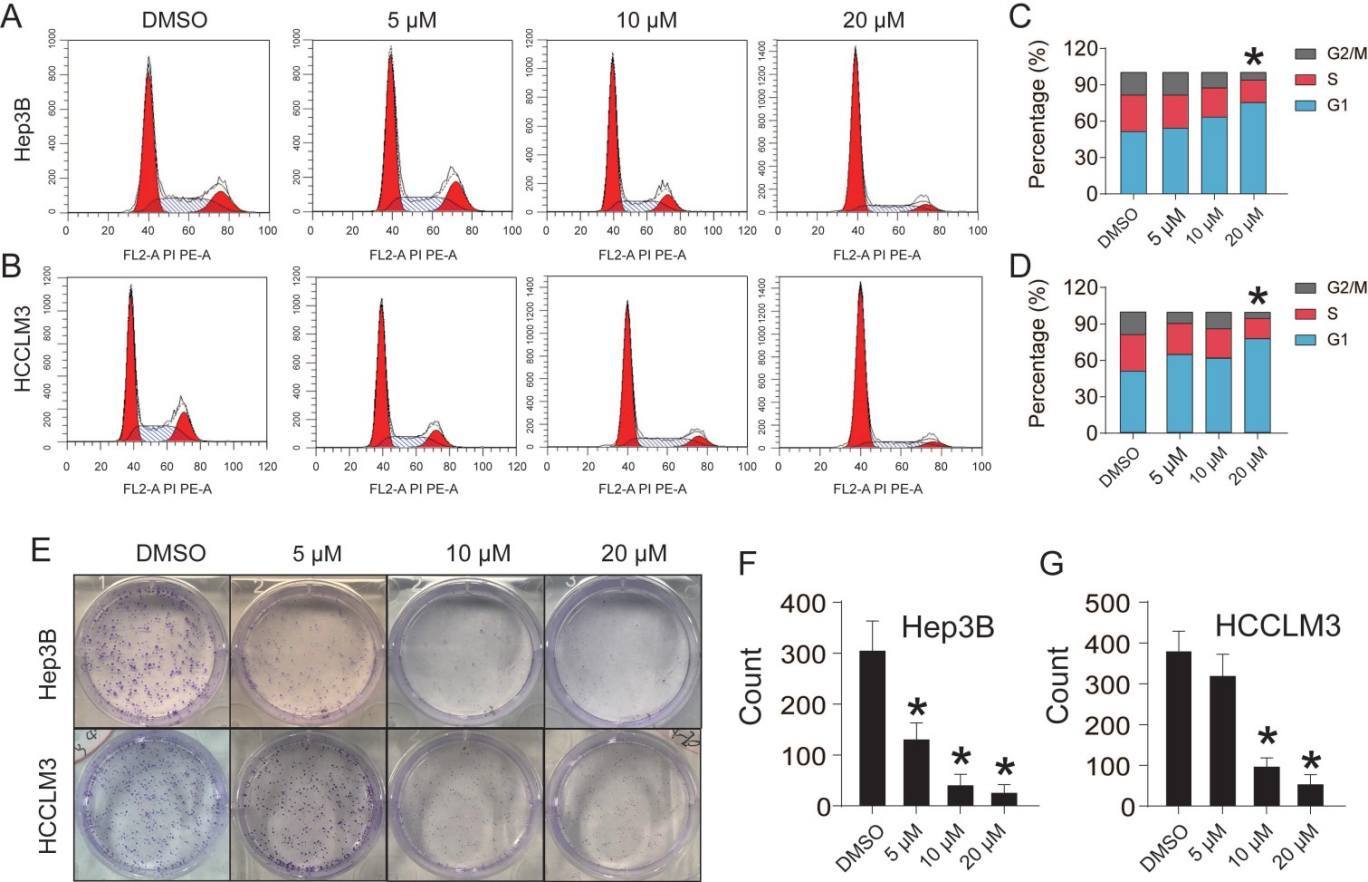
CEP suppresses Hep3B and HCCLM3 cell proliferation. (A, C) Cell cycle of Hep3B and HCCLM3 cells treated with CEP at 5 μM, 10 μM and 20 μM for 48h were analyzed by flow cytometry. (B, D) Percentage of indicated Hep3B and HCCLM3 cells in different phase. (E) Clone formation of Hep3B and HCCLM3 cells treated with CEP at 5 μM, 10 μM and 20 μM. (F, G) Quantification of the clone formation of Hep3B and HCCLM3 cells in (E) was performed by calculating the cell counts. Data shown are means ± SD; n=3. *P< 0.05, significantly from control group.

**Figure 3:**
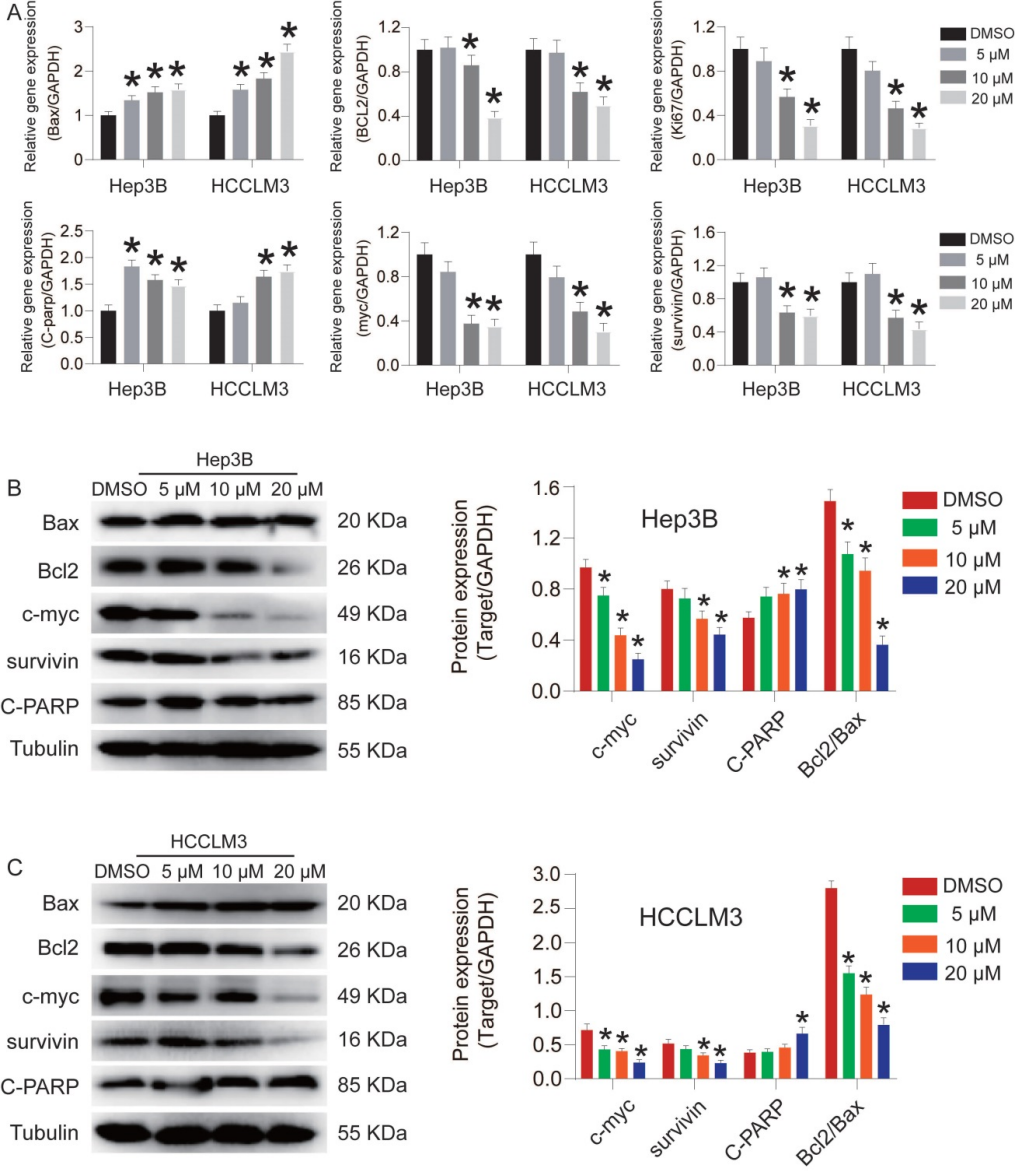
CEP inhibited the expression of Bax, Bcl2, Ki67, c-myc, survivin and C-PARP in HCC cells. (A) The gene expression of Bax, BCL2, Ki67, c-myc, surviving and C-PARP in Hep3B and HCCLM3 cells treated with CEP at 5 μM, 10 μM and 20 μM for 48h. The relative expression levels were analyzed by the 2-ΔΔct method. (B, C) The representative bands of Bax, BCL2, c-myc, surviving and C-PARP in Hep3B and HCCLM3 cells treated with CEP at 5 μM, 10 μM and 20 μM for 48 h. Tubulin was used as an internal control. Data shown are means ± SD; n=3. *P< 0.05, significantly from control group.

To investigate the effect of CEP on Hep3B and HCCLM3 cell proliferation, we used flow cytometry to detect changes in the cell cycle (Fig. 2A and 2B). The result showed that the number of Hep3B and HCCLM3 cells entering S phase was reduced by 9.01% and 8.68%, respectively, and the number of cells entering the G1 phase significantly increased after CEP treatment (20 µM) compared with those in the control group (Fig. 2C and 2D). Next, we found that CEP treatment led to a decrease of colony formation in HCC cells, indicating reduced oncogenicity (Fig. 2E). The number of colonies formed by Hep3B cells treated with 10 µM and 20 µM CEP decreased by 80.4% and 84.5%, respectively, and the number of colonies formed by HCCLM3 cells treated with CEP decreased by 48.7% and 59.3%, respectively, compared with the number in the control group treated with DMSO. In addition, the expression of proliferative protein c-myc in Hep3B and HCCLM3 cells treated with CEP for 48 h significantly decreased in a concentration-dependent manner (Fig. 3B). The mRNA expressions of c-myc and Ki67 were consistent with the protein expression trend (Fig. 3A). Therefore, these results indicated that CEP effectively inhibited the proliferation of Hep3B and HCCLM3 cells.

### CEP induces Hep3B and HCCLM3 cell apoptosis via the activation of caspase-9/3

**Figure 4:**
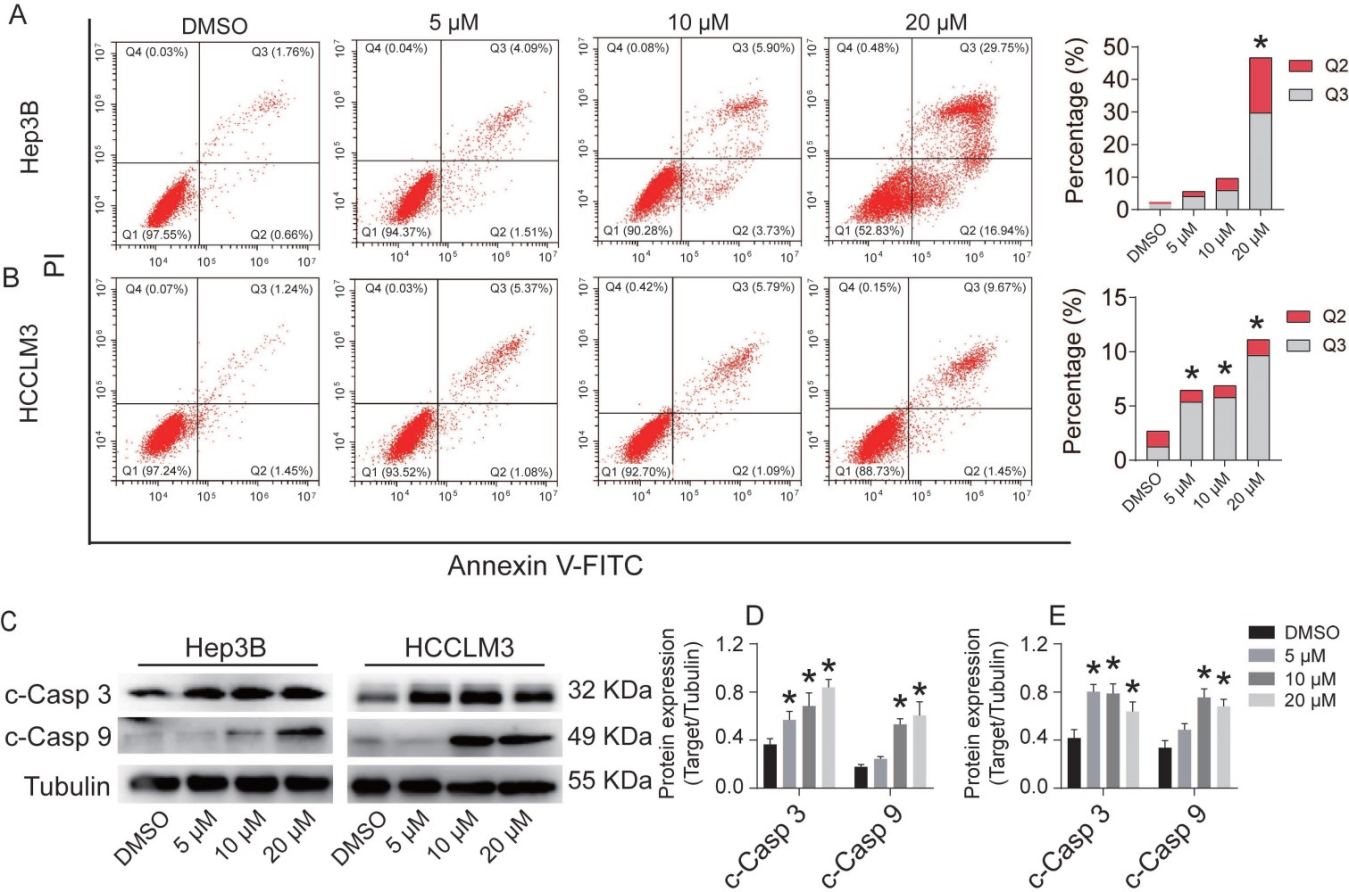
CEP induces Hep3B and HCCLM3 cells apoptosis via the activation of caspase-3/9. (A, B) Cell apoptosis of Hep3B and HCCLM3 cells treated with CEP at 5 μM, 10 μM and 20 μM for 48h were analyzed by flow cytometry. (C-E) The representative bands of c-caspase 3 and c-caspase 9 in Hep3B and HCCLM3 cells treated with CEP at 5 μM, 10 μM and 20 μM for 48h. Tubulin was used as an internal control. Data shown are means ± SD; n=3. *P< 0.05, significantly from control group.

Flow cytometry was used to detect the effect of CEP on Hep3B and HCCLM3 cell apoptosis (Fig. 4). After treatment with CEP for 48 h, the apoptosis rates of Hep3B cells in the DMSO group and 5 µM, 10 µM, and 20 µM groups were 2.42%, 5.60%, 9.63%, and 46.69%, respectively (Fig. 4A), whereas the apoptosis rates of HCCLM3 cells were 2.69%, 6.45%, 6.88%, and 11.12%, respectively (Fig. 4B). AnnexinV-FITC/PI apoptosis assay results showed that CEP could significantly promote the apoptosis of Hep3B and HCCLM3 cells. To further verify this result, we examined the changes of apoptosis-related protein and mRNA expressions. Western blotting and RT-PCR analysis demonstrated that CEP significantly reduced the expression levels of Bcl2 and survivin protein (Fig. 3A, 3B and 3C). Further experiments showed that the activation of caspase-3 and caspase-9 was significantly induced through its cleavage by CEP and the expression of cleaved-PARP was also up-regulated in a dose-dependent manner (Fig. 4C, 4D, and 4E). These results suggested that CEP induces Hep3B and HCCLM3 cell apoptosis through the activation of caspase-9/3.

### CEP inhibits Hep3B and HCCLM3 cell migration

**Figure 5:**
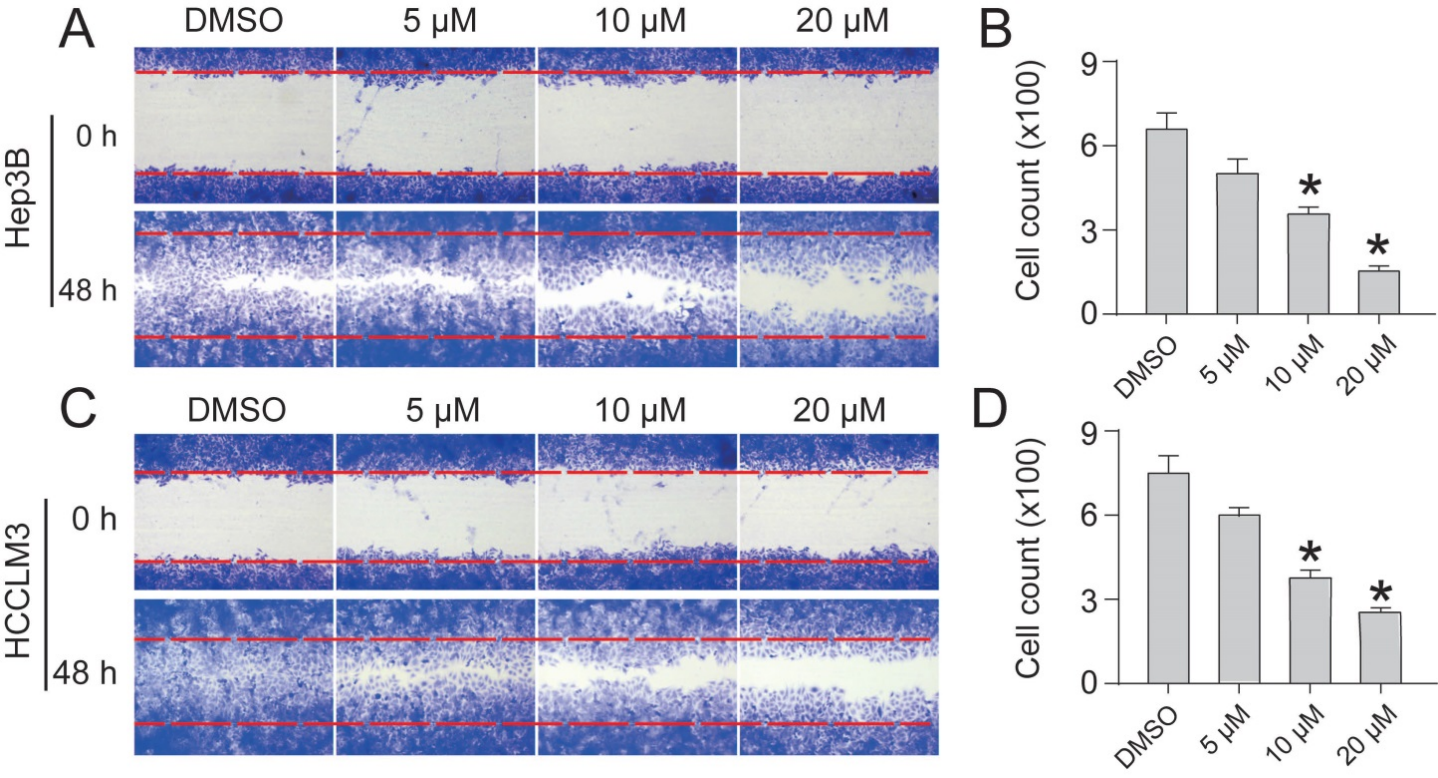
CEP inhibits Hep3B and HCCLM3 cells migration (A, C) Scratch assay was performed to determine migration of Hep3B and HCCLM3 cells treated with CEP at 5 μM, 10 μM and 20 μM for 48 h. (B, D) Quantification of the scratch image was performed by calculating the migrated cell counts in (A, C). Data shown are means ± SD; n=3. *P< 0.05, significantly from control group.

To assess the inhibitory effect of CEP on the migration of HCC cells, we performed a cell scratch assay. In the cell scratch assay, we found that CEP significantly inhibited the migration of Hep3B and HCCLM3 cells in a dose-dependent manner. Within 48 h, cells in the DMSO group began to migrate and almost completely filled the scratch area. However, compared with the control group, the CEP treatment group showed a gradual decrease in the counts of cell migration with increasing CEP con**ce**ntration. The migration of Hep3B and HCCLM3 cells was significantly reduced by 76.5% and 80.5% after 20 µM CEP treatment, respectively (Fig. 5).

### CEP regulates differentially expressed genes and pathway enrichment analysis

**Figure 6:**
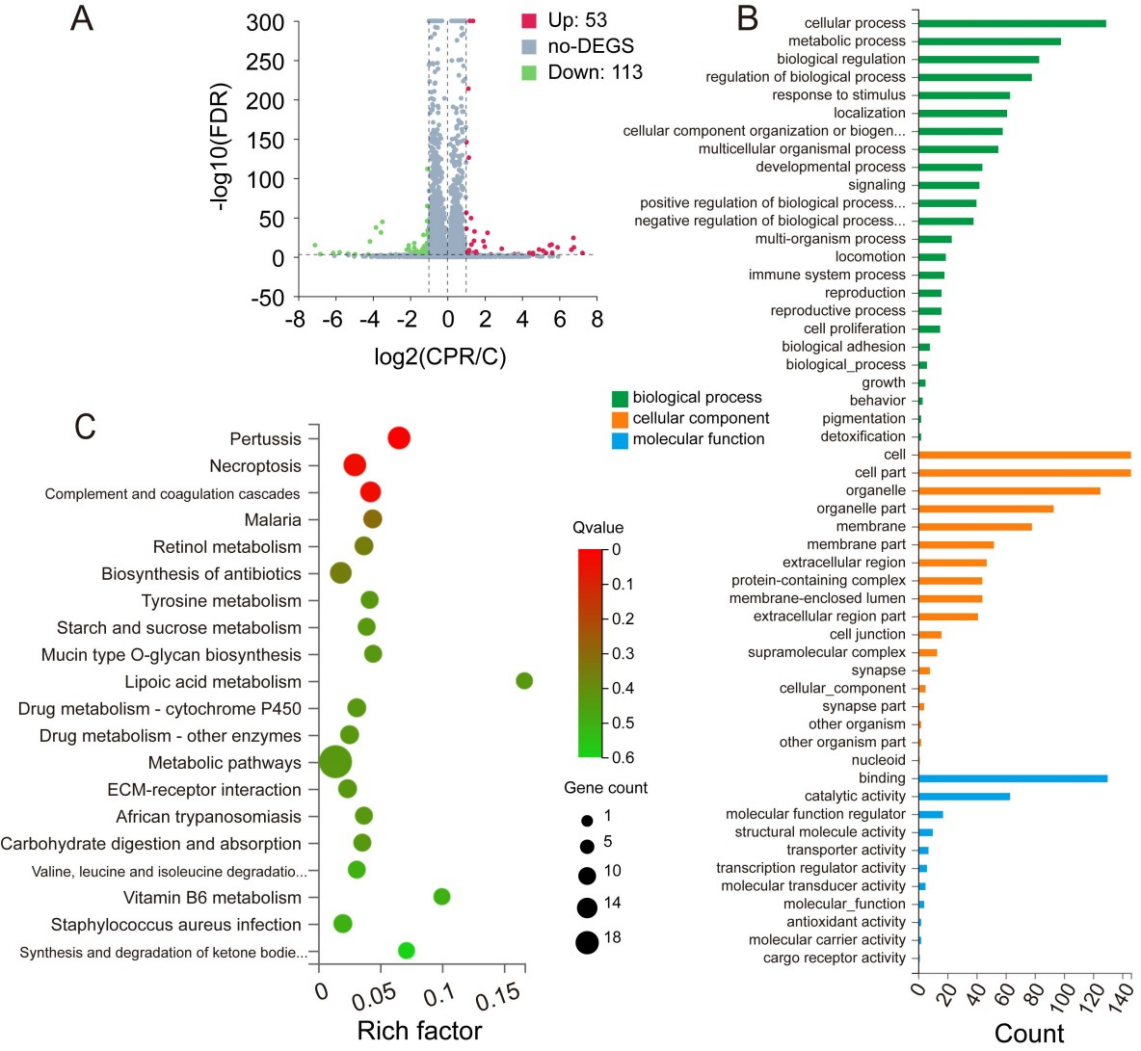
CEP regulates differentially expressed genes and pathway enrichment analysis (A) Significant differentially expressed genes were shown in volcano plot. FC (fold change) > 1 was accepted as positive differentially expressed genes, up for 53; down for 113. (B) GO annotations analysis of HCCLM3 cells treated with CEP, compared with control group. (C) KEGG pathway enrichment analysis, a larger P value (-Log10) shows a higher degree of enrichment. Significant pathways involving in necroptosis and metabolic pathways of HCCLM3 cells treated with CEP.

Compared with the control group, RNA-Seq results revealed that the expression of 53 genes was up-regulated, whereas that of 113 genes was down-regulated after CEP treatment. The number of down-regulated genes was significantly higher than that of up-regulated genes, which proved that CEP had an obvious inhibitory effect on gene transcription and made the basic function of cells stagnated (Fig. 6A). GO annotation of differentially expressed genes (DEGs) revealed the regulation of cellular and metabolic processes in biological process (BP) classification. Cellular component (CC) classification of DEGs was significantly enriched in intracellular membrane-bounded organelle and cell part. Molecular function (MF) classification of DEGs showed significant enrichment in binding and catalytic activity (Fig. 6B). Further, the KEGG signal pathway enrichment showed that these DEGs were highly enriched in necroptosis and metabolic-associated pathways (Fig. 6C).

### CEP adjusts the amino acid metabolism pathway in HCCLM3 cells

**Figure 7:**
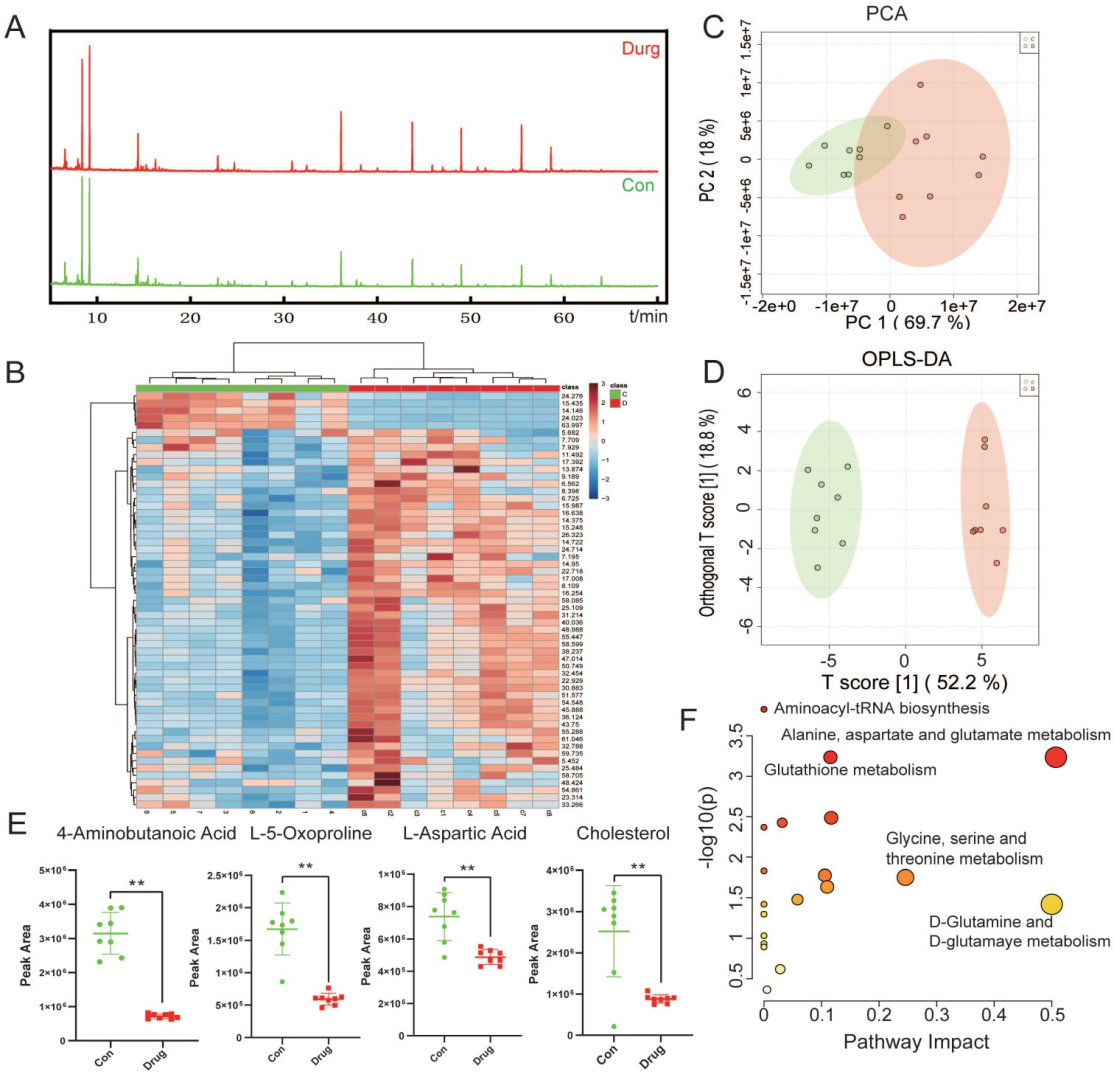
CEP regulates the amino acid metabolism pathway in HCCLM3 cells (A) The base peak chromatogram of metabolites between Control and Drug (20 μM CEP) group. (B) The PCA score plot of Control and Drug group, it represents samples in the groups were closely cluster to one another. (C) The OPLS-DA score plot of Control and Drug group revealed the clustering of samples in the training set. (D)Heat map of metabolites showing between Control and Drug group. Increased metabolites were marked in red. Decreased metabolites levels were presented in blue. (E) Metabolites related to lipid metabolism altered by CEP treatment in HCCLM3 cells. n=10 replicates per group. (F) Plots depict the computed metabolic pathways as a function of -log (p) (y-axis) and the pathway impact of the key metabolites (x-axis) that differed between the Control and Drug group. The color of a circle is indicative of the level of enrichment significance, with yellow being low and red being high. The size of a circle is proportional to the pathway impact value of the pathway.

Abnormal metabolic changes are an important feature of malignant tumors and play an important role in the development of tumors. The results of KEGG signaling pathway enrichment showed that the DEGs were enriched in the metabolic pathway. Accordingly, we used the metabonomic method for further verification. GC-MS was used to evaluate the effect of CEP treatment on the primary metabolites of HCCLM3 cells (Fig. 7A). The heat map shows that CEP can affect the production of many primary metabolites in the cell (Fig. 7B). The principal component analysis score diagram and orthogonal partial least square discriminant analysis were used to evaluate sample quality by differentiating between groups. The differences between the control and drug groups were significant, and the differences were relatively concentrated within the group, indicating that the differences within the group were small after drug intervention (Fig. 7C and 7D). Further analysis showed that the expression of the following four metabolites, namely, 4-aminobutanoic acid, L-5-oxoproline, L-aspartic acid, and Cholesterol (Fig. 7E) was significantly reduced in the 20 µM CEP treatment group. Furthermore, metabolic pathway analysis showed that these differential metabolites are mainly enriched in amino acid metabolism (glutathione metabolism; alanine, aspartate, and glutamate metabolism; glycine, serine, and threonine metabolism; and D-glutamine and D-glutamate metabolism) (Fig. 7F), suggesting that CEP can play an anticancer role by regulating amino acid metabolism.

### CEP suppresses transplanted tumor growth *in vivo*

**Figure 8:**
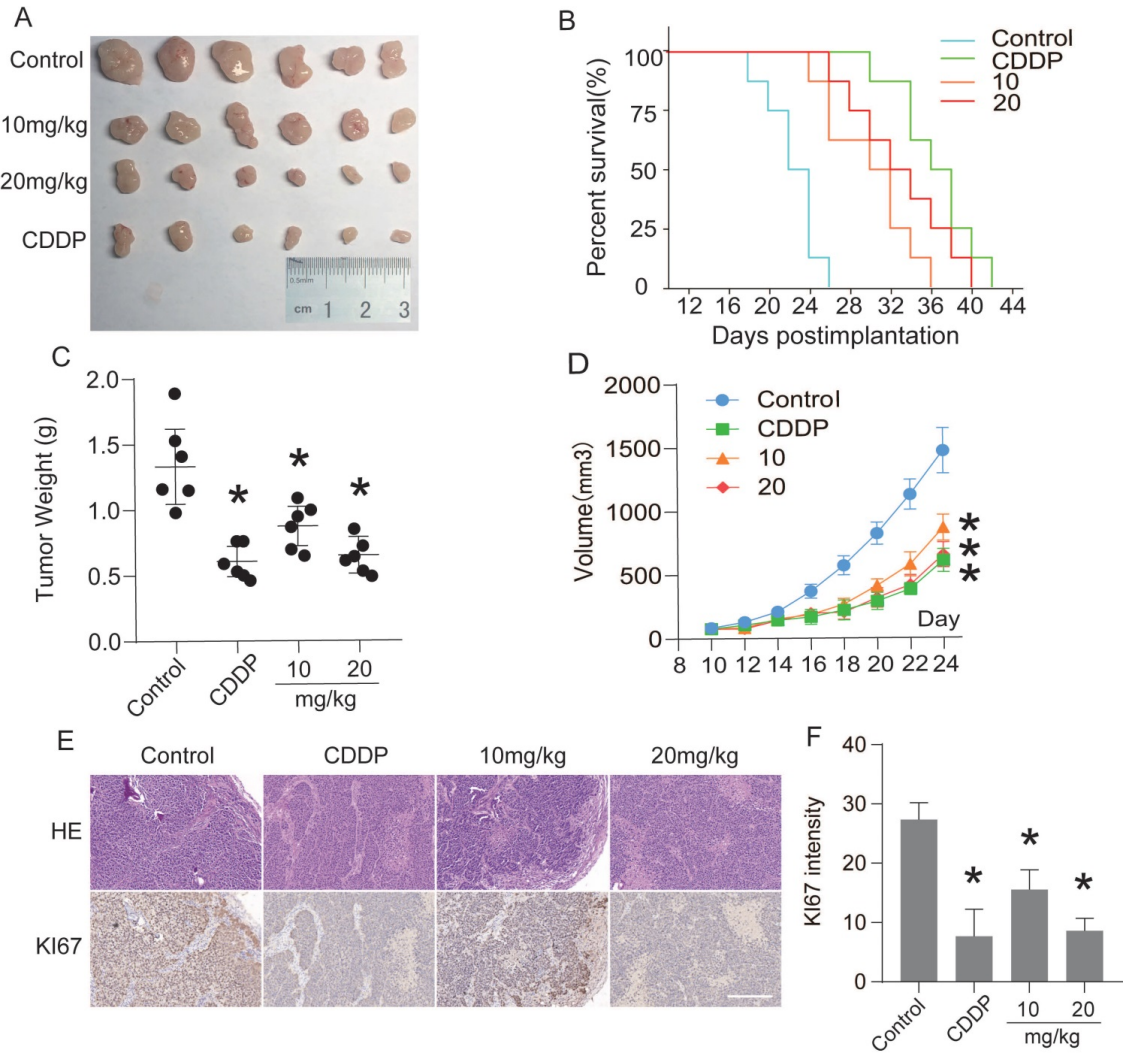
CEP suppressed transplanted tumor growth *in vivo*. Twenty-four nude mice were randomly divided into negative control group, positive control group (CDDP, 5 mg/kg, i.p., once two days), low-concentration treatment group (CEP, 10 mg/kg, i.p., once two days) and high-concentration treatment group (CEP, 20 mg/kg, i.p., once two days). (A) Photograph of the tumors. (B) Survival curve of indicated mice, mice with tumors larger than 1000 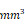 are considered dead. (C, D) Tumor weight and volume of indicated mice. (E) H&E staining and IHC of KI67 in indicated tumors. (F) Quantitative image analysis of KI67 in (E). Scale bar=100 μm. Data shown are means ± SD; n=3. *P< 0.05, significantly from control group.

The antitumor effect of CEP was evaluated by subcutaneous tumor transplantation in nude mice. Ten days after Hep3B cells were injected into the armpits of nude mice, the mice were randomly divided into negative control group, positive control group (CDDP, 5 mg/kg, i.p., once in two days), low-concentration treatment group (CEP, 10 mg/kg, i.p., once in two days), and high-concentration treatment group (CEP, 20 mg/kg, i.p., once in two days). After 14 days of CEP treatment, the tumor weight and volume in CEP and CDDP treatment groups were significantly lower than those in the negative control group (DMSO treatment group) (Fig. 8A, 8C and 8D). The survival curve in each treatment group showed that the median survival time of the negative control, positive control, low concentration treatment, and high-concentration treatment groups were 22 days, 36 days, 30 days, and 32 days, respectively, indicating that CEP treatment significantly prolonged the survival of nude mice with transplanted tumors (Fig. 8B). These results showed that CEP treatment could markedly inhibit the growth of transplanted tumors *in vivo*. To further analyze the antitumor effect of CEP, we performed H&E staining and immunofluorescence staining with Ki67 on tumor samples. Compared with the negative control group, the number of cells in the CEP treatment group was significantly reduced (Fig. 8E). Further, immunohistochemical results showed that the expression of the proliferation marker protein Ki67 decreased with an increase in CEP concentration, and the effect of 20 mg/kg CEP was similar to that of CDDP (Fig. 8E and 8F). All animal experiments showed that CEP significantly suppressed tumor growth *in vivo*.

## Discussion

Liver cancer is one of the most common causes of cancer-related deaths worldwide, and is characterized by a high malignancy rate and poor prognosis. Because of the multiple causes of HCC, its rapid development, and late diagnosis, less than 15% of HCC patients can receive surgical resection. Therefore, chemotherapy and immunotherapy are the best options for HCC treatment. Currently, there are only a few commonly used chemotherapy drugs for liver cancer. However, with the long-term use of many chemotherapy drugs, such as sorafenib, side effects such as toxicity and efficacy decrease may occur 5. Therefore, developing drug candidates and therapeutic targets for the treatment of liver cancer is necessary and urgent.

With the exploration of antioxidant and anti-tumor functions of natural plant compounds, many active extracts of natural medicinal plants have been used in cancer treatment and have shown good anti-tumor efficacy. Piperine, extracted from black pepper, for example, has anti-tumor, anti-oxidation, and anti-proliferative activities, and it can activate the expressions of caspase3 and caspase9 in HepG2 cells and promote the apoptosis of liver cancer cells 22. Alantolactone, extracted from the root of artichoke, regulates cell cycle arrest and activates the mitochondrial apoptosis pathway in liver cancer cells 23. A number of other natural compounds can be used in combination therapy to reduce the side effects of chemotherapy or enhance therapeutic efficacy; for example, allium atroviolaceum bulb extract has a synergistic effect with doxorubicin 24 and 5-Fu has an enhanced tumor-inhibiting effect in combination with matsutake polysaccharide 25. CEP is a naturally occurring small molecular compound extracted from the plant *Stephania cepharantha* Hayata. It is used to treat a variety of acute and chronic diseases, such as malaria 26, alopecia 27, and endotoxic shock 28. Recent studies have shown that CEP also has anti-tumor effects, and has an inhibitory effect on breast cancer 29, lung cancer 30, and esophageal squamous cell carcinoma 31. CEP therapy inhibits autophagy and mitochondrial autophagy by blocking autophagosome-lysosomal fusion in human breast cancer cells 29. In this study, we discussed the anti-tumor effect of CEP on hepatocellular carcinoma. We provide evidence that CEP has potential as a candidate drug for HCC treatment.

Studies have shown that one of the important features of cancer is the excessive proliferation of tumor cells caused by abnormal cell cycle, thus inhibiting the cell cycle of tumor cells is considered as an attractive method to treat cancer 32. We demonstrated that CEP treatment can reduce the activity of HCC cells, inhibit their ability to proliferate and migrate, and regulate the cell cycle. C-myc is a pleiotropic transcription factor that regulates the cell cycle, proliferation, and growth. Studies have shown that c-myc is associated with the occurrence, development, and prognosis of liver cancer 33, 34. Our results suggest that CEP can inhibit the proliferation of HCC cells by inhibiting c-myc expression.

In addition, our transcriptome sequencing results showed that DEGs in the CEP treatment group were enriched in metabolic pathways. Abnormal metabolic changes play an important role in the survival and development of tumors, supporting the proliferation of cancer cells by measures such as increasing energy supply and maintaining redox balance 35. Tumor metabolism involves glucose metabolism, amino acid metabolism, lipid metabolism, and many other phenomena. Inhibition of cancer cell metabolism is a current tumor treatment goal 36. In the liver, the synthesis of non-essential amino acids plays an important role in maintaining function. Alterations in amino acid metabolism are characteristics of HCC 37, 38. Our subsequent validation experiments confirm that CEP could inhibit the metabolism of HCC cells. Metabonomic results have shown that CEP treatment decreased the production of 4-aminobutanoic acid, L-5-oxoproline, and L-aspartic. Among these, aspartic acid is an essential substance for the synthesis of nucleotides *in vivo* 39. Tumor cells need a large amount of aspartic acid for the synthesis of nucleotides to promote tumor growth. Studies have shown that under hypoxic conditions, reducing the level of aspartic acid can inhibit tumor cell growth 40. Abnormal cholesterol metabolism in tumor cells leads to increased cholesterol synthesis and uptake, leading to proliferation, invasion, and metastasis of tumor cells 41. Studies have found that elevated mitochondrial cholesterol content in HCC leads to reduced mitochondrial membrane permeability and increases chemotherapy resistance of tumor cells 42. Our results suggest that CEP can reduce cholesterol levels and inhibit the cholesterol metabolism in HCC cells, indicating that CEP can inhibit the metabolism of tumor cells by regulating amino acid metabolism and cholesterol metabolism and thus inhibit the growth of tumor cells.

Cell death can be divided into programmed and non-programmed death according to whether or not it is controllable 43. Apoptosis is the most typical and most studied form of programmed cell death. Apoptosis is mainly induced by the cysteine protease family, in which caspase-2/8/9/10 are the initiator caspases and caspase-3/6/7 are effector Caspases 44. Zhu et al. showed that CEP can induce cell apoptosis in melanoma cells by regulating apoptosis-related proteins, including Bcl-2 and caspase 45. Our flow cytometry results suggest that CEP promotes cell death by promoting the apoptosis of HCC cells. Further protein and mRNA expression verification indicated that CEP could induce apoptosis through the Bax/Bcl2/Caspase-9/Caspase-3 signaling pathway (Fig. 4). In recent years, another newly discovered programmed cell death mode, necroptosis, has become a hot research topic 46. Necroptosis is affected by receptor-interacting protein kinase 1 (RIPK1), receptor-interacting protein kinase 3 (RIPK3), and mixed lineage kinase domain-like protein (MLKL) 47. In our study, transcriptome sequencing and KEGG signaling pathway enrichment data indicated that CEP could regulate necroptosis of HCC cells.

Furthermore, *in vivo* subcutaneous tumor-formation experiments in nude mice confirmed that CEP could significantly inhibit the growth of solid tumors. Here, our study showed that intraperitoneal injection of CEP (10 or 20 mg/kg, once in two days) could significantly inhibit the weight and volume of Hep3B-induced hepatocellular carcinoma *in vivo* and prolonged the survival time of nude mice with transplanted tumors (Fig. 8). The immunohistochemical and immunofluorescence tests showed that the anti-tumor effect of CEP was related to the inhibition of expression of Ki67, which is used as a marker of tumor proliferation (Fig. 8D).

## Conclusion

In summary, our available data demonstrates that CEP can inhibit the proliferation of HCC cells by regulating cellular metabolism through amino acid metabolism and cholesterol metabolism. At the same time, CEP can inhibit tumor growth by promoting apoptosis and necrosis and inducing the death of HCC cells. Therefore, CEP may be a candidate anticancer drug for the treatment of HCC.

## Figures and Tables

**Table 1:** Sequences of the Real-time qPCR.

RT-qPCR Primers		
Gene Name	**Forward**	**Reverse**
Bcl-2	GTGGCCTTCTTTGAGTTCG	CATCCCAGCCTCCGTTAT
Bax	CCCGAGAGGTCTTTTTCCGAG	CCAGCCCATGATGGTTCTGAT
Ki67	AGCCCGTATCTGGTGCAAAA	CCTGCATCTGTGTAAGGGCA
myc	TACAACACCCGAGCAAGGAC	TACAACACCCGAGCAAGGAC
Survivin	AGGACCACCGCATCTCTACAT	AAGTCTGGCTCGTTCTCAGTG
C-PARP	TCCAGCAGGCGGTGTCTCAG	CTCGATGTCCAGCAGGTTGTCAAG
GAPDH	CATCACGCCACAGTTTCC	ATCATCAGCAATGCCTCC

## Abbreviations

CEP: Cepharanthine
HCC: Hepatocellular carcinoma
GC-MS: Gas chromatography mass spectrometric
DEGs: differentially expressed genes
CDDP: Cisplatin
BCL2: B-cell lymphoma 2
FBS: fetal bovine serum
DMSO: dimethyl sulfoxide
CCK-8: cell counting kit-8
PBS: pre-cooled phosphate buffer
BCA: bicinchoninic acid
PVDF: polyvinylidene fluoride
Bax: BCL-2 associated X
C-PARP: cleaved poly ADP-ribose polymerase
Caspase: cysteinyl aspartate specific proteinase
HRP: horseradish peroxidase
ECL: enhanced chemiluminescence
H&E: hematoxylin and eosin
DAB: 3,3'-Diaminobenzidine
SD: standard deviation
